# Clinical features and diagnostic value of metagenomic next -generation sequencing in five cases of non-HIV related *Pneumocystis jirovecii* pneumonia in children

**DOI:** 10.3389/fcimb.2023.1132472

**Published:** 2023-03-16

**Authors:** Jiechao Niu, Jiandong Wang, Peisheng Jia, Mengjiao Zhang, Erhu Wei

**Affiliations:** Department of Pediatrics, The First Affiliated Hospital of Zhengzhou University, Zhengzhou, China

**Keywords:** clinical features, MNGs, diagnosis, infection, NH-PJP

## Abstract

**Background:**

*Pneumocystis jirovecii* (PJ) is an opportunistic pathogenic fungus, and PJ pneumonia (PJP) is a commonly problem in HIV-positive patients. While PJP is not caused by HIV, it generally advances rapidly and can quickly lead to severe respiratory failure. To improve pediatricians’ understanding of the condition and aid early accurate diagnoses and therapy, we examined the clinical characteristics of five instances of non-HIV related PJP (NH-PJP) in children and the efficacy of metagenomic next-generation sequencing (mNGS) in its diagnosis.

**Methods:**

From January 2020 to June 2022, five children with NH-PJP were admitted to the PICU of the First Affiliated Hospital of Zhengzhou University. We retrospectively summarize the clinical presentation, previous histories, routine laboratory findings, treatment, outcome of regression, and results of mNGS in these five children.

**Results:**

Five male children between the ages of 11 months and 14 years had an acute onset on NH-PJP, three of the children had chest tightness after activity, shortness of breath and paroxysmal dry cough, — and two had high fever and dry cough. All five of the children had several flocculent high-density pictures in both lungs at the beginning of the disease, and lung auscultation revealed coarse breath sounds in both lungs, one of which was accompanied by a modest quantity of dry rales. PJ nuclear sequences were found in one patient and four patients’ blood and alveolar lavage fluid. All five children were treated with Trimethoprim-sulfamethoxazole (TMP-SMX) in combination with Caspofungin and corresponding symptomatic treatment. Four patients were cured and one patient died.

**Conclusion:**

Children commonly encounter an initial exposure to NH-PJP, which manifests as a high fever, dry cough, chest discomfort, dyspnea that worsens over time, fast disease progression, and a high death rate. The clinical presentation of children with PJ infection should be taken into consideration along with the results for diagnose. mNGS has higher sensitivity and a shorter detection period compared to identification of PJP.

## 1 Introduction

*Pneumocystis jirovecii* (PJ) is an opportunistic pathogenic fungus, and PJ pneumonia (PJP) is commonly found in HIV-positive patients. With the use of chemotherapy or immunosuppressive medication in recent decades, the prevalence of non-HIV related PJ (NH-PJP) has been rising annually (Catherinot et al., 2010; Avino et al., 2016). The clinical presentation of PJP varies greatly between HIV-infected and non-HIV-infected patients (Bienvenu et al., 2016; Liu et al., 2019). In the HIV- infected population, patients with PJP usually present with sub-acute onset of gradual dyspnea, nonproductive or minimally productive cough, low grade fever and malaise. Early in the course of infection patients may be asymptomatic (Oladele et al., 2018). NH-PJP progresses rapidly and is difficult to diagnose correctly, thus leading to severe respiratory failure (Cordonnier et al., 2016; Liu et al., 2019). NH-PJP mortality rates have been reported to range from 28% to 53%, which is significantly higher compared to HIV-infected patients (17% to 30%) (Cordonnier et al., 2016). NH-PJP symptoms include high fever, dry cough, chest pain, and progressively worsening dyspnea with oxygen and impairment. Patients with NH-PJP often present with symmetrical diffuse ground glass shadows in both lungs, along with patchy shadows, interstitial alterations, and air sac-like changes on computed tomography (CT) scan (McKinnell et al., 2012).

The prognosis for children with NH-PJP depends greatly on early and timely diagnosis, precise anti-PJP treatment, and awareness of the seriousness and rapid progression of NH-PJP (Lu et al., 2022). In recent years, a molecular biology technique called metagenomic next-generation sequencing (mNGS) has been developed, which has the potential to identify more than 15,000 pathogenic microorganisms with known genome sequences (Gu et al., 2019). However, there are few clinical examples of applying bronchoalveolar lavage fluid (BALF) mNGS (BALF-mNGS) to diagnose PJP. Herein, we have retrospectively analyzed the clinical data of five children with NH-PJP and the results of mNGS examination. To increase pediatricians’ comprehension of the condition and aid assist, as well as to promote prompt and accurate treatment, we summarize and evaluate the clinical characteristics of NH-PJP in children and discuss the diagnostic utility of mNGS technology in confirming PJP.

## 2 Methods

### 2.1 Study design and participants

In this retrospective study, we consecutively enrolled PJP patients who were admitted to the PICU of the First Affiliated Hospital of Zhengzhou University, from January 2020 to June 2022. Patients were eligible for enrollment if they met all the following criteria: (1) immunocompromised conditions, including but not limited to hematologic malignancies, solid tumors, rheumatic diseases, long-term systemic use of corticosteroids (0.3 mg/kg/day of prednisone equivalent for > 3 weeks), use of immunosuppressive agents (including chemotherapeutic agents for malignancies, but not corticosteroids), solid organ transplantation and hematopoietic stem cell transplantation;(2) typical clinical manifestations of PJP, including fever, cough, dyspnea, and progressive hypoxemia; (3) radiologic findings suggestive of PJP in bilateral lungs that newly emerged on CT scans; (4) BALF and/or blood samples were collected for mNGS. Furthermore, we collected patients’ baseline information, clinical features, laboratory and imaging examination results, diagnoses, treatments, and outcomes. Patients were excluded if they met any of the following criteria: (1) age≥18 years old; (2) HIV infection; (3) mNGS was not performed; (4) medical record was incomplete.

### 2.2 mNGS protocol

#### 2.2.1 Sample processing and sequencing

First 3-4mL of blood was drawn from patients, placed in EDTA tubes, and stored at room temperature for 3–5 minutes before plasma separation and centrifuged at 1,600 g for 10 min at 4°C within 8 hours of collection. Plasma samples were transferred to sterile tubes. DNA was extracted from 300 μL of plasma using the TIANamp Micro DNA Kit (DP316, TIANGEN BIOTECH, Beijing, China) following the manufacturer’s instructions. The extracted DNA specimens were used for the construction of DNA libraries.

BALF was collected based on the standard clinical procedure. Briefly, 20 mL saline was injected into a segmental bronchus and drawn back after a while. Next, 3 mL of BALF was inactivated at 65°C for 30 minutes immediately after collection. Subsequently, a 1.5-mL microcentrifuge tube with 0.5-mL sample and 1 g 0.5-mm glass beads were attached to a horizontal platform on a vortex mixer and agitated vigorously at 2800–3200 prm for 30 min. Finally 0.3-mL sample was collected into a new 1.5-mL microcentrifuge tube and DNA was extracted using the TIANamp Micro DNA Kit (DP316, TIANGEN BIOTECH) according to the manufacturer’s instructions.

According to the protocol of the BGISEQ-50 sequencing platform, the DNA library was constructed through DNA fragmentation, end-repair, adapter-ligation, and PJR amplification. The constructed library was qualified by Agilent 2100 (Agilent Technologies, Santa Clara, CA, USA) and Qubit 2.0. The qualified double-strand DNA library was transformed into a single-stranded circular DNA library through DNA-denaturation and circularization. DNA nanoballs (DNBs) were generated from single-stranded circular DNA using rolling circle amplification (RCA). The DNBs were qualified using Qubit 2.0. Qualified DNBs were loaded into the flow cell and sequenced (50 bp, single-end) on the BGISEQ-50 platform.

#### 2.2.2 Bioinformatic analysis

High-quality sequencing data were generated by removing low-quality and short (length < 35 bp) reads using in-house software, followed by computational subtraction of human host sequences mapped to the human reference genome (hg19) using Burrows–Wheeler Alignment. After the removal of low-complexity reads, the remaining data were classified by simultaneously aligning to four Microbial Genome Databases, consisting of viruses, bacteria, fungi, and parasites. The four Microbial Genome Databases were downloaded from NCBI (ftp://ftp.ncbi.nlm.nih.gov/genomes/). RefSeq contains 4,061 whole-genome sequences of viral taxa, 2,473 bacterial genomes or scaffolds, 199 fungi, and 135 parasites associated with human diseases. The number of unique alignment reads was calculated and standardized to get the number of reads stringently mapped to pathogen species (SDSMRN) and the number of reads stringently mapped to pathogen genus (SDSMRNG).

## 3 Results

### 3.1 General information

**Figure 1:**
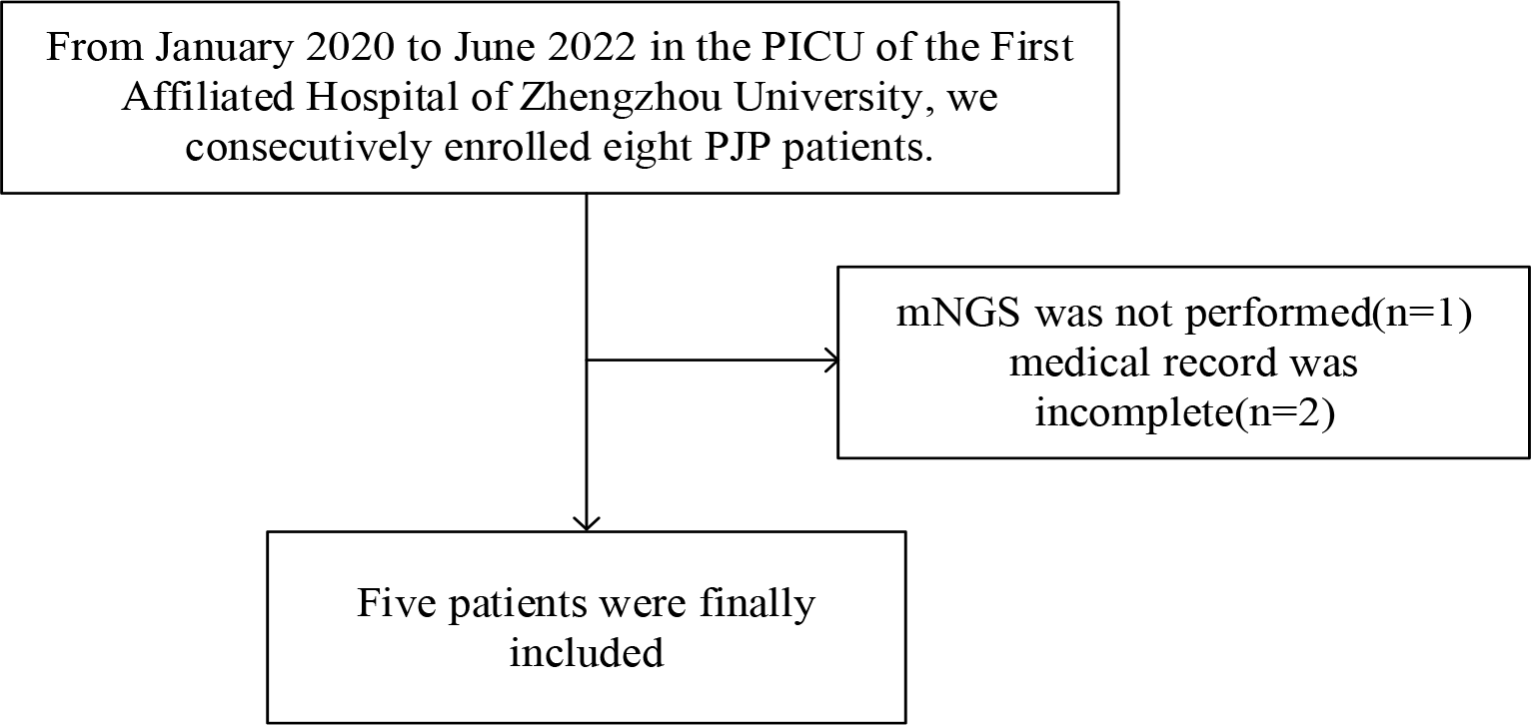
Flow diagram of the patients included in the study.

In this study, five children were eventually enrolled (**Figure 1**).

Case 1, male, 3 years old, with underlying disease of autoimmune encephalitis, was treated with rituximab (375 mg/m^2^, two doses) and regular maintenance therapy with prednisone (10 mg/d) during the treatment of the underlying disease.
Case 2, male, 11 months, with acute lymphoblastic leukemia as the underlying disease, was treated with the PVDL regimen (Prednisone, Vincristine, Daunorubicin and L-Asparaginase) at presentation.
Case 3, male, 8 years old, was in a post-transplant state and was taking tacrolimus regularly.
Case 4, male, 14 years old, in post-renal transplant status, on regular Tacrolimus.
Case 5, male, 6 years old, with underlying disease of special sarcoma, treated with VDC regimen (Vincristine, Cyclophosphamide and Doxorubicin) before the onset of the disease. five children had no abnormal personal, growth and developmental, or family history.

### 3.2 Clinical manifestations and signs

Cases 1, 4, and 5 started with chest tightness after activity, shortness of breath, and paroxysmal dry cough, and developed fever with a fever peak of 39°C or higher on the 2nd_4th day of illness. Cases 2 and 3 started with fever peaking at 39°C with dry cough. Five children developed respiratory distress within 3_4 days after the onset of the disease and were mechanically assisted with ventilation on day 3 in case 1, on day 10 in case 2 and on day 7 in cases 3, 4 and 5. In case 2, coarse breath sounds and a few dry rales could be heard in both lungs at the onset of the disease, and in the other four children, coarse breath sounds could be heard in both lungs as far as possible.

### 3.3 Laboratory test results

Peak LDH (233_2002) U/L, peak BDG (10.00_2470.6) pg/mL, and CD4 lymphocyte count (18.32_985)/μL in five children (**Table 1**).

**Table 1:** The results of CRP, PCT, LDH, BDG and CD4 lymphocyte count in five children.

NO.	peak of CRP(0-5mg/L)	peak of PCT(0-0.2ng/L)	peak of LDH(200-600U/L)	peak of BDG(0-95pg/mL)	CD4 lymphocyte count(550-1440/uL)
Case1	42.95	2.03	2002	584.32	985
Case2	90.23	5.84	233	<10	NA
Case3	35.66	0.864	174	100.37	840
Case4	101	4.83	502	254.28	282.26
Case5	51.34	0.237	834	2470.6	18.32

NA, Not Answer.

### 3.4 Chest imaging results

**Figure 2:**
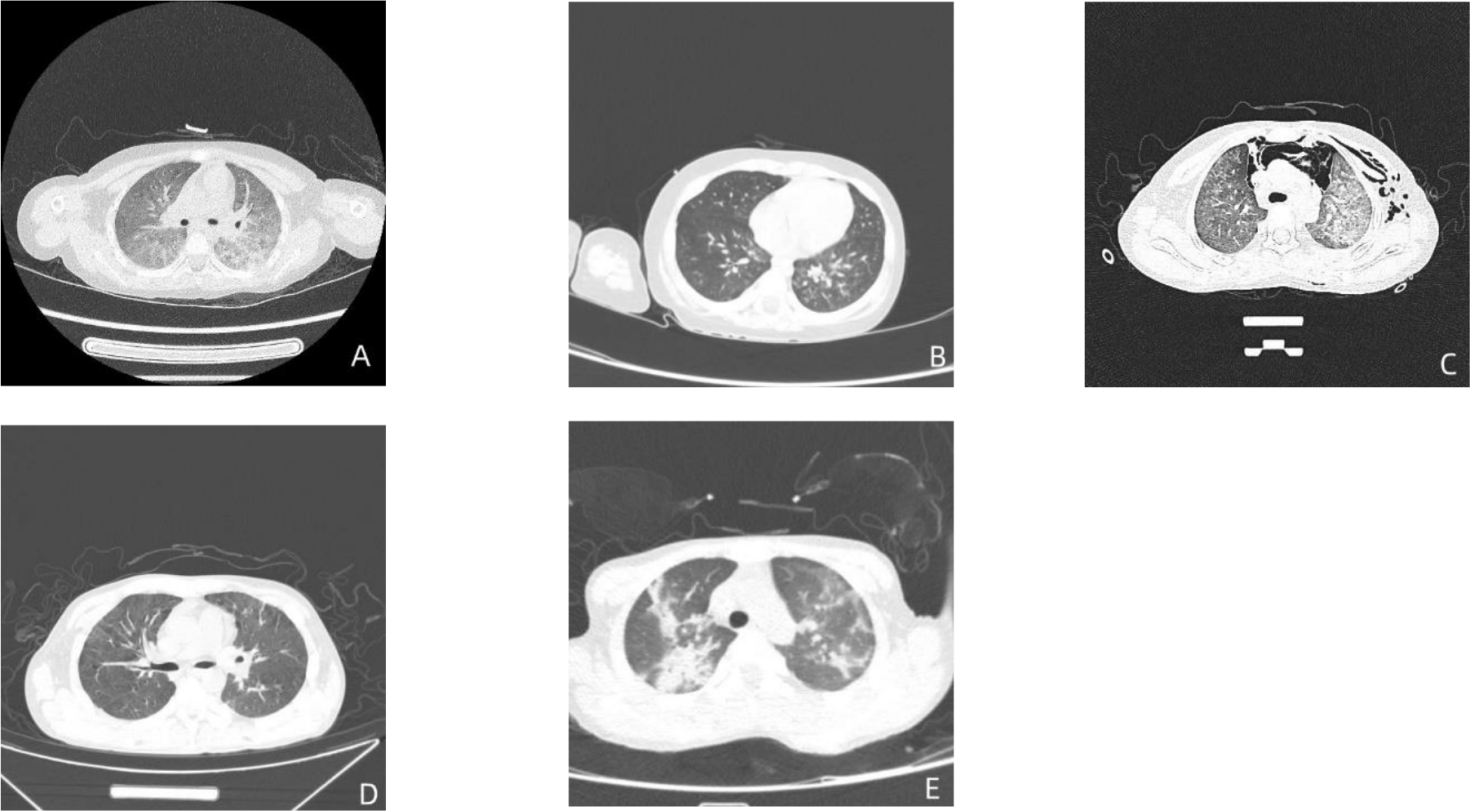
Pulmonary CT results in five children. The figure **(A–E)** are the CT images of the lungs of case 1-5 in order.

All five children showed multiple flocculent high-density images in both lungs on chest CT (**Figure 2**).

### 3.5 mMGS and pathology test results

#### 3.5.1 mNGS test results

The five children had gene sequence numbers of 340, 3, 1797, 27025, and 63156, respectively, with mNGS showing positive PJP. These children were co-infected with other pathogens, such as Streptococcus pneumoniae and cytomegalovirus (**Table 2**).

**Table 2:** Results of mNGS in five children.

NO.	Sample of mNGS	Number of gene sequences	Relative abundance	Combination of other pathogens
Case 1	BALF	340	69.11%	*S. pneumoniae* Cytomegalovirus Herpes simplex virus
Case 2	Blood	3	100%	*S. pneumoniae*
Case 3	BALF	1797	89.19%	*S. pneumoniae* Cytomegalovirus
Case 4	BALF	27025	3.16%	Deficient oxygen-depleted bacteria
Case 5	BALF	163156	96.79%	Klebsiella pneumoniae Acinetobacter baumannii Haemophilus influenzae

#### 3.5.2 Regie’s stain results

Case 1 and case 4 results suggest positive, case 3 and case 5 results suggest negative, case 2 was not tested.

**Figure 3:**
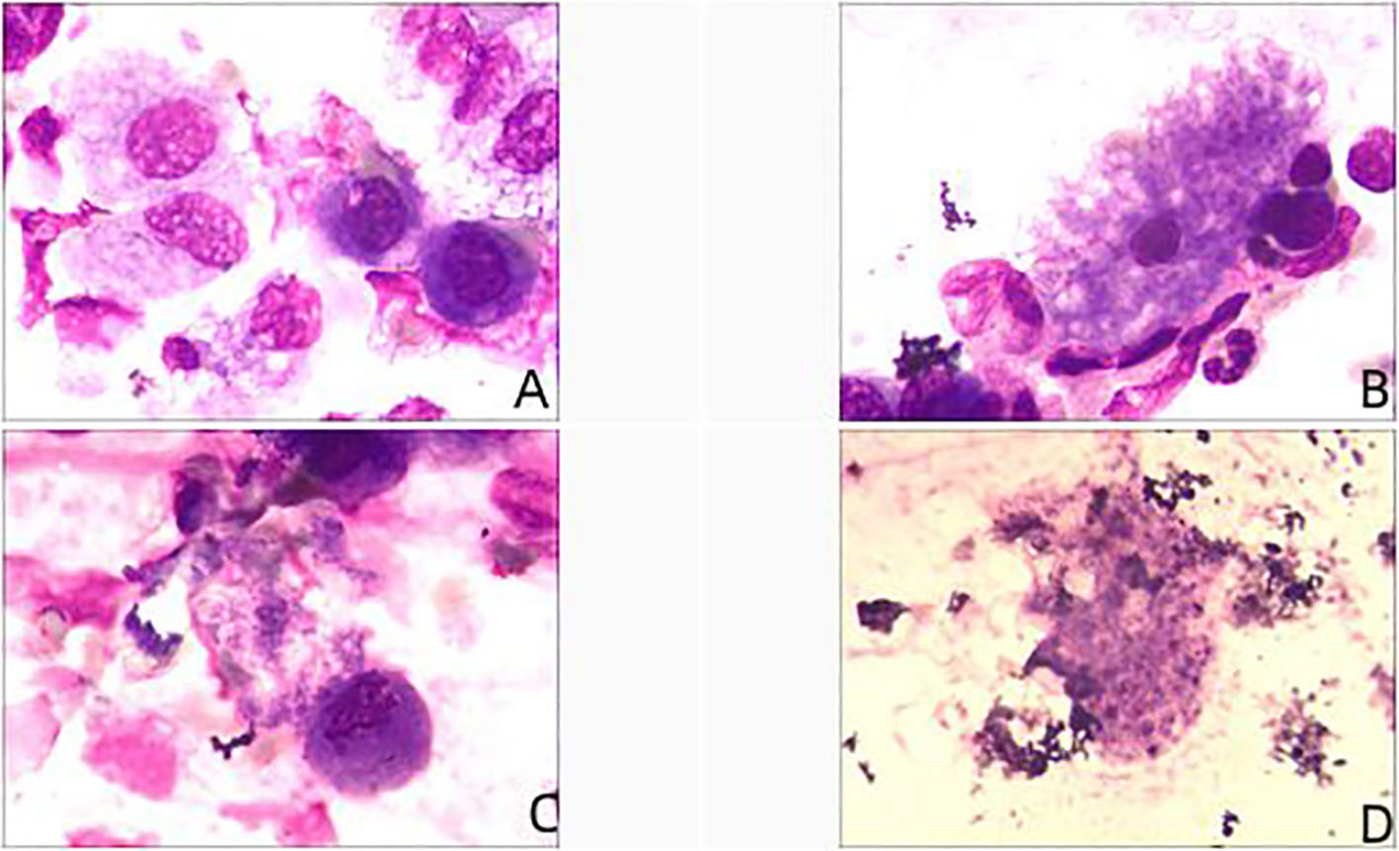
Regie’s stain results of case 1 **(A, B)** and case 4 **(C, D)**.

PJ and encapsulation were seen in the BALF of case 1 (**Figures 3A, B**) and case 4 (**Figures 3C, D**).

#### 3.5.3 Fungal immunofluorescence staining results

No fungus was seen under fungal immunofluorescence staining in all four children (case 2 was not tested).

### 3.6 Treatment history and regression

Cases 1, 3, 4 and 5 were transferred to the PICU of the First Affiliated Hospital of Zhengzhou University within 3 to 4 days after the onset of illness without targeted treatment, and were treated immediately with TMP-SMX combined with caspofungin anti-infective therapy, alveolar lavage and mechanical assisted ventilation support. Among them, cases 1, 3 and 4 were treated with methylprednisolone, and cases 1 and 4 were treated with intravenous immunoglobulin. All four children recovered. In case 2, blood mNGS was checked on the 3rd day after fever, which tested positive for *Enterococcus faecalis* and *Streptococcus vestibularis*. After 9 days of combined treatment with meropenem and teicoplanin and 3 days of caspofungin treatment, the child still had an intermittent fever and respiratory distress, and the oxygen saturation was maintained at about 90% by transdermal oxygen measurement under 5 L/min of face mask inhalation. Case 1, 3, 4 and 5 were cured and Case 2 was dead (**Table 3**).

**Table 3:** Treatment and outcome of five children.

NO.	Time of starting treatment	Time of drug treatment	Improvement time after medication	Outcome
Case1	3	19	12	Cured
Case2	11	1	NA	Dead
Case 3	4	20	13	Cured
Case 4	4	20	13	Cured
Case 5	3	21	14	Cured

NA, Not Answer.

## 4 Discussion

*In vitro* cultivation of unusual fungus, PJ, is extremely challenging. Traditionally, diagnosis relies on the presence of specific encapsulated or trophoblast cells in specimens such as BALF or sputum, radiological manifestations, and clinical symptoms (Bateman et al., 2020). Despite being simple and inexpensive, traditional microscopic examination is less sensitive for PJP because of the low fungal load. Despite having 100% specificity for the diagnosis of PJP, immunofluorescence and cytological staining only exhibited a 74% and 50% sensitivity, respectively, according to a meta-analysis (Senécal et al., 2022). In this study, PJ was detected among alveolar lavage fluid in only two children due to the limited sensitivity of the conventional assay. Because of the significant limits of the conventional approach, a more efficient and sensitive assay is required to fulfill clinical demands.

PJP-induced lung inflammation and damage can result in an aberrant rise in LDH (Oladele et al., 2018). LDH has been confirmed to be a reliable non-specific marker for PJP, with a sensitivity of 96% and a specificity of 77% when LDH >379 U/L (Sun et al., 2021). Due to the limited sample size in this study, only three children had LDH levels over 379 U/L, which meant that we were unable to clearly establish the significance of LDH in the diagnosis of NH-PJP. Moreover, further large-scale studies are required to confirm whether LDH can indicate the severity of PJP disease and the degree of lung inflammation.

DG is a polysaccharide synthesized from D-glucan, which is a specific component of the fungal cell wall, accounting for more than 50% of the fungal cell wall composition. DG testing can be used as a diagnostic aid in patients at high risk for invasive fungal disease, especially in patients with blood disorders (Lu et al., 2011a; Lu et al., 2011b). It has a specificity of 75% and a sensitivity of 90% for the diagnosis of PJP (Del et al., 2020).

In this study, case 2 was tested for BDG level only upon disease onset; he was not examined again throughout the consultation. Further, BDG levels were checked many times throughout the consultation in the other four instances. In cases 1, 4, and 5, the BDG levels were significantly elevated, further suggesting the possibility of PJP infection. According to the European Conference on Infections in Leukemia (ECIL), a negative serum BDG is sufficient to exclude PJP; however, a positive serum BDG is not specific for the diagnosis of PJP, necessitating further tests to confirm the diagnosis (Maschmeyer et al., 2016).

In addition, despite the great sensitivity of the BDG test, it can still give false positive results in cases of hemodialysis, gram-negative bacteremia, severe mucositis or intravenous immunoglobulin and certain antibiotics. Therefore, the BDG level alone cannot be used to diagnose PJP (Li et al., 2015). Clinical manifestations and additional laboratory test results must be paired with the PJP diagnosis in order to confirm it (Mercier et al., 2019).

Polymerase chain reaction (PCR) for PJ was initially developed in the 1980s with primers against the pneumocystis mitochondrial large-subunit ribosomal gene (Dunbar et al., 2020). PCR has been shown to be more sensitive for detection of PJP compared to staining methods in patients with and without HIV (Lu et al., 2011a; Lu et al., 2011b). However, mNGS outperformed the conventional method (i.e., *in vitro* culture, PCR) in the detection of MTB, bacteria, fungi, mycoplasma, and viruses (Huang et al., 2021).

The advantages in mNGS over traditional detection methods include a shorter detection cycle, a wider detection range, and a higher positive rate. This makes mNGS particularly suitable for conditions such as pathogenic bacteria that cannot be clarified by traditional detection methods or for clinical aspects that require rapid clarification of pathogenic bacteria. In comparison with BDG and hexamine silver staining, mNGS showed a sensitivity of 100% and a specificity of 96.3% in the diagnosis of PJP, according to research by Jiang et al. (2021). Liu et al. assessed the effectiveness of serum BDG and BALF-mNGS in aiding the diagnosis of PJP in their study (Liu et al., 2021). BDG >88.6 pg/mL and mNGS sequence number >14 exhibited 90% sensitivity and 14% specificity in diagnosing PJP. mNGS can assist in the diagnosis of PJP more efficiently and accurately than infectious immunofluorescent staining (Liu et al., 2021; Lu et al., 2022).

However, case 2 tested negative when the blood sample was tested on day 5 after the onset of the disease and only tested positive when the blood sample was tested again for mNGS on day 10 after disease onset. In four of the five children in our study, PJ was measured when BALF was tested within 3_5 days after the onset of the disease. This phenomenon suggests that BALF should be utilized as the test sample as much as possible in the clinical use of mNGS for the diagnosis of PJP. If only the blood specimen can be tested and the clinical manifestations and laboratory findings are highly suggestive of PJP, PJP cannot be excluded because of the first negative result, and mNGS should be performed at least once, but ideally more times. In addition, it is important to keep in mind that the microbial sequences reported by mNGS include potentially pathogenic organisms. As a result, the clinical setting must be properly considered when interpreting mNGS data. Although the list can help to qualify the range of pathogenic organisms, mNGS cannot yet accurately determine whether the flora is colonized or infected.

PJ was detected in all five patients using mNGS with a sensitivity of 100%, whereas when PJ and encapsulation were discovered using pathological staining and microscopy on tissues from four patients, the sensitivity was only 50%. The mNGS experimental cycle is shorter than the pathology assay, with data available in 48 hours as opposed to the pathology assay cycle, which is typically 3 days, and the mNGS assay was more sensitive in this study. The pathology results are more influenced by the technician’s experience, while it is not necessary for the technician to be familiar with PJ for mNGS to simultaneously identify and compare all microbial genomes in a sample for analysis. The diagnosis PJ infections can be greatly aided using mNGS, which can also help doctors to quickly identify pathogenic organisms and administer prompt therapy to enhance the effectiveness of their interventions.

Several limitations of the study should be mentioned. First, the major limitation of this study is the small number of patients, due to the rarity of the disease. Second, this is a single center retrospective study; thus, intrinsic bias was unavoidable. Third, diagnostic performances of mNGS and PCR were not compared in this study because PCR of PJ was not routinely performed in our hospital. This line of query should be explored in future work.

In conclusion, to lessen the burden of NH-PJP patients, there is an urgent need for improved preventive and treatment measures. This is supported by the epidemiology, morbidity, and mortality of NH-PJP. Patients with suspected PJP should start anti-PJP specific medication immediately to prevent treatment delays brought on by diagnostic procedures such as bronchoalveolar lavage, which increases the need for mechanical assisted ventilation and disease mortality. The most crucial element in reducing NH-PJP related mortality is a straightforward, precise, and easily accessible diagnostic approach. The combination of BALF-mNGS should be used clinically whenever possible to improve the detection rate of NH-PJP, but the results should be interpreted with a comprehensive consideration of the actual clinical situation because mNGS is a new microbiological assay with some advantages over traditional assays in identifying Pneumocystis.

## Data availability statement

The original contributions presented in the study are publicly available. This data can be found here: https://www.ebi.ac.uk/ena/browser/view/PRJEB59277.

## Ethics statement

The studies involving human participants were reviewed and approved by The First Affiliated Hospital of Zhengzhou University. Written informed consent to participate in this study was provided by the participants’ legal guardian/next of kin.

## Author contributions

JN and JW collected the original clinical data and processed the statistical data. JN drafted and edited the manuscript. PJ and MZ participated in the design, and EW revised the manuscript. All authors contributed to the article and approved the submitted version.
