# Erratum: Clinical features and diagnostic value of metagenomic next -generation sequencing in five cases of non-HIV related *Pneumocystis jirovecii* pneumonia in children

**DOI:** 10.3389/fcimb.2023.1233206

**Published:** 2023-06-14

**Authors:** Frontiers Production Office

**Affiliations:** Frontiers Media SA, Lausanne, Switzerland

**Keywords:** clinical features, MNGs, diagnosis, infection, NH-PJP

Due to a production error, the Data Availability Statement that was originally published was incorrect.

The Data Availability Statement is:

“The original contributions presented in the study are publicly available. This data can be found here: https://www.ebi.ac.uk/ena/browser/view/PRJEB59277”.

The publisher apologizes for this mistake.

The original version of this article has been updated.

