# Supplementary figures and images for: Clinical features and diagnostic value of metagenomic next -generation sequencing in five cases of non-HIV related Pneumocystis jirovecii pneumonia in children
Source: Front Cell Infect Microbiol. 2023 Mar 16;13:1132472. doi: 10.3389/fcimb.2023.1132472 (PMC10060869; doi:10.3389/fcimb.2023.1132472)

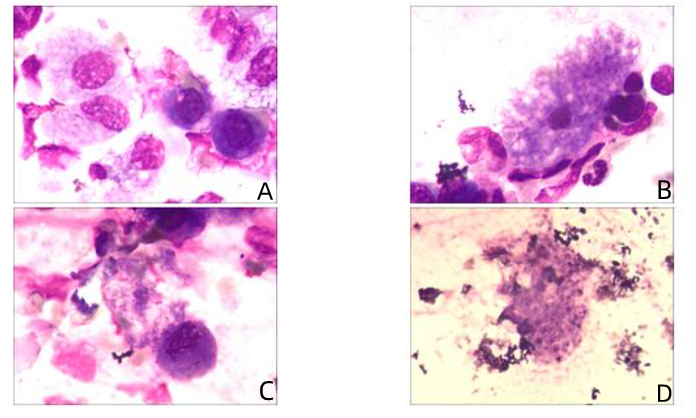

Supplement: Supplementary file 1 [file Image_1.png]

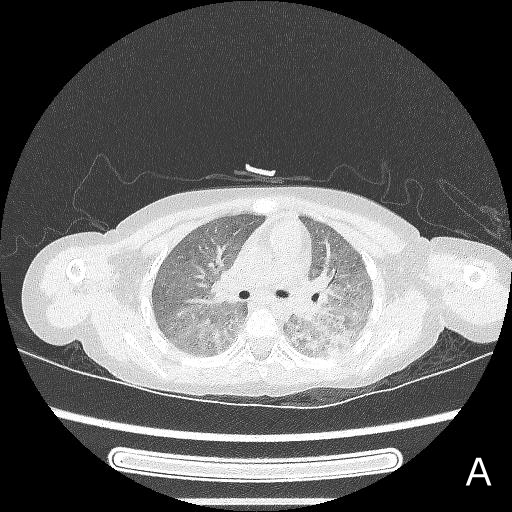

Supplement: Supplementary file 2 [file Image_2.jpeg]

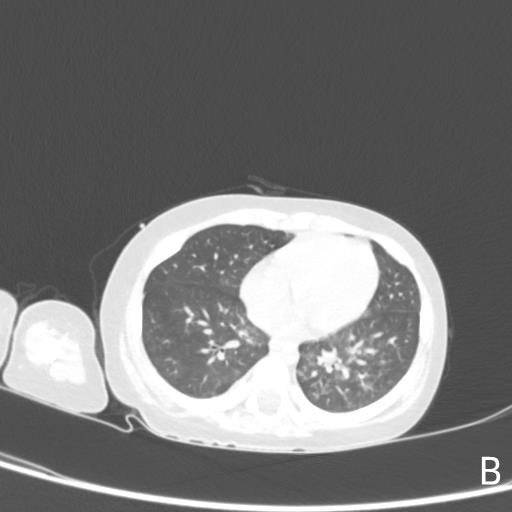

Supplement: Supplementary file 3 [file Image_3.jpeg]

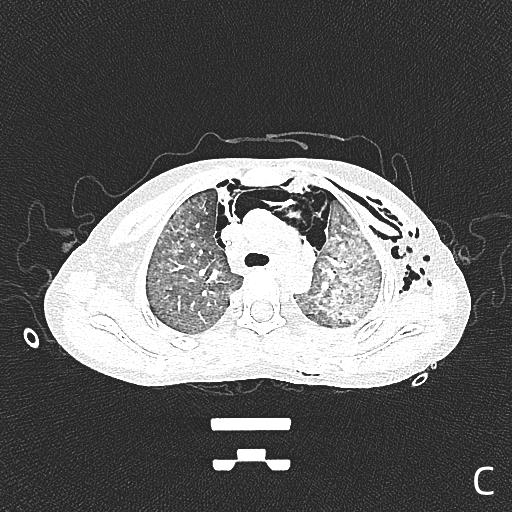

Supplement: Supplementary file 4 [file Image_4.jpeg]

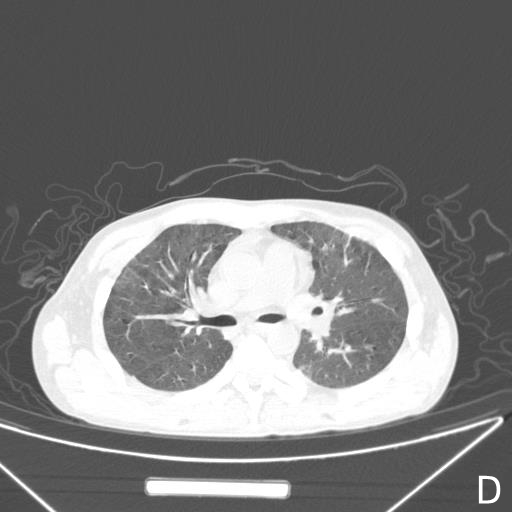

Supplement: Supplementary file 5 [file Image_5.jpeg]

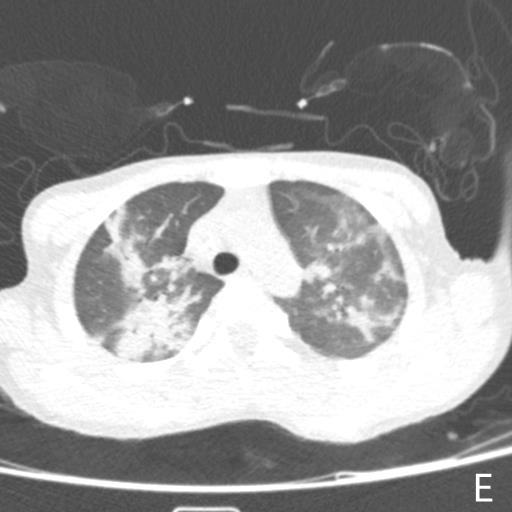

Supplement: Supplementary file 6 [file Image_6.jpeg]
